# Immunotherapy in the First-Line Treatment of NSCLC: Current Status and Future Directions in China

**DOI:** 10.3389/fonc.2021.757993

**Published:** 2021-11-25

**Authors:** Anwen Xiong, Jiali Wang, Caicun Zhou

**Affiliations:** ^1^ Department of Medical Oncology, Shanghai Pulmonary Hospital, Thoracic Cancer Institute, Tongji University School of Medicine, Shanghai, China; ^2^ Medical Research Lab (MRL) Global Medical Affairs, MSD China, Shanghai, China; ^3^ Department of Medical Oncology, Shanghai Pulmonary Hospital, Tongji University School of Medicine, Shanghai, China

**Keywords:** non-small cell lung cancer (NSCLC), immunotherapy, immune checkpoint inhibitors (ICI), pembrolizumab, China

## Abstract

Lung cancer causes significant morbidity and mortality in China and worldwide. In China, lung cancer accounts for nearly one-fourth of all cancer deaths. Non-small cell lung cancer (NSCLC) is the predominant type of lung cancer, accounting for approximately 80%–85% of all lung cancer cases. Immunotherapy with immune checkpoint inhibitors (ICIs) is revolutionizing the treatment of NSCLC. Immune checkpoint molecules, including PD-1/PD-L1 and CTLA-4, can suppress immune responses by delivering negative signals to T cells. By interfering with these immunosuppressive axes, ICIs unleash antitumor immune responses, ultimately eliminating cancer cells. ICIs have demonstrated promising antitumor efficacy in NSCLC, and mounting evidence supports the use of ICIs in treatment-naïve patients with advanced NSCLC. A comprehensive overview of current and emerging ICIs for the first-line treatment of NSCLC in China will facilitate a better understanding of NSCLC immunotherapy using ICIs and optimize the clinical use of ICIs in previously untreated Chinese patients with NSCLC. Herein, we review the efficacy and safety of currently approved and investigational ICIs as the first-line treatment of NSCLC in China. We also discuss the challenges limiting more widespread use of ICIs and future directions in the first-line treatment of NSCLC using ICIs.

## Introduction

Lung cancer is the leading cause of cancer-related death in China, accounting for 23.8% of all cancer deaths according to the GLOBOCAN 2020 epidemiological data (1). The incidence of lung cancer in China has increased drastically in recent years, posing a significant threat to human health and placing a considerable financial burden on the Chinese healthcare system (2–6). According to the most recent data from the National Cancer Center of China, over 774,000 new cases of lung cancer were diagnosed in 2018, approximating 18% of all newly diagnosed cancers (7). In the same year, over 690,000 patients died due to lung cancer (7). In contrast to the increasing incidence of lung cancer in China, the incidence and mortality associated with lung cancer in the US and UK has been decreasing since the 1990s, likely due to the decrease in smoking prevalence in these countries (8). Furthermore, the age-standardized mortality rate of lung cancer in China is 1.4 times higher than the corresponding mortality rates in the USA (7).

The vast majority of lung cancers are diagnosed at a locally advanced or metastatic stage, contributing to the low 5-year survival rate (9–11). Lung cancers are broadly classified as non-small cell and small cell lung cancer. Non-small cell lung cancer (NSCLC) accounts for 80%–85% of all lung cancer cases (10). NSCLCs are further classified based on the cell type of origin into adenocarcinoma (nearly 66%), squamous cell carcinoma (nearly 28%) and other classified or rare subtypes accounting for approximately 6% of all NSCLC cases (12).

Cancer immunotherapy is developing rapidly, and multiple immunotherapy options are available for the first-line treatment of NSCLC in China. Immune checkpoint inhibitors (ICIs) are the most promising immunotherapies for NSCLC. Immune checkpoint molecules, such as programmed cell death protein 1 (PD-1)/programmed death-ligand 1 (PD-L1) and cytotoxic T-lymphocyte-associated protein 4 (CTLA-4), deliver negative signals to T cells, preventing destructive overactivation of the immune system and conferring immunological homeostasis (13). Hence, by blocking immune checkpoint molecules, ICIs unleash antitumor T cell responses, which, in turn, eliminate cancer cells (14).

Despite the promising antitumor effects of ICIs in patients with NSCLC, there are currently no articles summarizing the efficacy and safety of approved and emerging ICIs for the first-line treatment of NSCLC in China, hindering a better understanding of NSCLC immunotherapy and improvement of immunotherapy outcomes in patients with NSCLC. Therefore, in this article, we comprehensively review current immune checkpoint blockade (ICB) strategies for the first-line treatment of NSCLC in China. We also discuss future directions in the first-line treatment of NSCLC using ICIs.

## Current Treatment Landscape For Advanced NSCLC in China

### Standard NSCLC Treatment

The treatment of patients with lung cancer in China is usually based on the pathologic and genetic status of the tumor (15). Surgical resection remains the treatment of choice for patients with early-stage NSCLC; however, postoperative recurrence rates are high, contributing to poor long-term survival outcomes (9, 16). Other conventional therapies commonly used to treat patients with NSCLC include radiotherapy, platinum-based chemotherapy regimens (e.g., pemetrexed plus cisplatin), and the combination of chemotherapy with radiotherapy or antiangiogenic agents (e.g., bevacizumab) (9, 15–17). Nevertheless, the long-term survival outcomes of patients with NSCLC who receive radiotherapy or chemotherapy are unsatisfactory (9).

The clinical implementation of molecular targeted therapies, such as tyrosine kinase inhibitors (TKIs) targeting the epidermal growth factor receptor (EGFR) mutation, anaplastic lymphoma kinase (ALK) rearrangement, and ROS1 rearrangement, etc., has improved the survival outcomes of patients with NSCLC harboring driver mutations (9, 15, 18, 19). However, patients with NSCLC lacking driver mutations do not benefit from targeted therapies (20, 21). ICIs, adoptive T cell therapies, cytokine-induced killer cells, and other emerging immunotherapies are transforming the treatment of NSCLC, especially those lacking driver mutations (9, 22–26).

### Current NSCLC Immunotherapy Landscape

The development of immunotherapies, and particularly of antibodies inhibiting CTLA-4 and PD-1/PD-L1 pathways, has revolutionized the treatment of NSCLC and improved the survival outcomes of patients with advanced or metastatic NSCLC in China and worldwide. Despite the fact that immunotherapies have significantly improved the long-term survival outcomes for patients with various tumor types, the development of primary resistance to immunotherapies remains a clinical challenge limiting the clinical benefit of ICIs to only a small proportion of patients (27). Among ICIs, those targeting the PD-1/PD-L1 axis are the most promising, exerting potent antitumor effects by reversing T cell exhaustion and unleashing antitumor immune responses (28, 29). Most immuno-oncology trials in China have focused on the clinical use of anti-PD-1 and anti-PD-L1 antibodies; the efficacy and safety of agents targeting other immunomodulatory molecules, such as TIGIT and LAG3, are also under investigation (30–32).

For patients with advanced, driver oncogene-negative NSCLC, several PD-1/PD-L1 inhibitors have gained regulatory approval in China as first-line treatment, alone or in combination with chemotherapy. China National Medical Products Administration (NMPA)-approved first-line ICIs for advanced NSCLC include pembrolizumab (alone in patients whose tumors express PD-L1 or in combination with pemetrexed/platinum for nonsquamous NSCLC and carboplatin/paclitaxel for squamous NSCLC), atezolizumab (alone in patients whose tumors express PD-L1), camrelizumab (combined with pemetrexed/carboplatin for nonsquamous NSCLC), sintilimab (combined with pemetrexed/platinum for nonsquamous NSCLC and platinum/gemcitabine for squamous NSCLC), and tislelizumab (combined with pemetrexed/platinum for nonsquamous NSCLC or with carboplatin/paclitaxel or nab-paclitaxel for squamous NSCLC) (**Table 1**). In addition to pembrolizumab and atezolizumab, which were developed by multinational corporations (55, 56), three locally developed PD-1 inhibitors (camrelizumab, tislelizumab, and sintilimab) are also available in China (31, 48, 57–59).

**Table 1:** Summary of approval status, indications and supporting clinical trials for immune therapies that are approved for use in patients with NSCLC.

Drug name	Trial supporting regulatory approval	Indication	NMPA approval	FDA approval
Pembrolizumab (MSD)	KEYNOTE-189 (33)	In combination with pemetrexed and platinum chemotherapy as a first-line treatment for patients with metastatic non-squamous NSCLC, with no EGFR or ALK genomic tumor aberrations	**✔**	**✔**
	KEYNOTE-042 (34–36)	As monotherapy for the first-line treatment of patients with locally advanced or metastatic NSCLC expressing PD-L1 (Tumor Proportion Score ≥1%), with no EGFR or ALK genomic tumor aberrations	**✔**	**✔**
	KEYNOTE-407 (37, 38) and KEYNOTE-407 China extension (39)	In combination with carboplatin and paclitaxel^a^ as first-line treatment for patients with metastatic squamous NSCLC	**✔**	**✔**
Atezolizumab (Roche)	IMpower110 (40, 41)	As monotherapy for the first-line treatment of patients with metastatic NSCLC whose tumors have high PD-L1 expression (PD-L1 stained ≥50% of tumor cells or PD-L1 stained tumor-infiltrating immune cells covering ≥10% of the tumor area), with no EGFR or ALK genomic tumor aberrations	**✔**	**✔**
	IMpower 150 (42, 43)	In combination with bevacizumab, paclitaxel, and carboplatin, for the first-line treatment of adult patients with metastatic non-squamous NSCLC with no EGFR or ALK genomic tumor aberrations	✖	**✔**
	IMpower 130 (44, 45)	In combination with albumin-bound paclitaxel and carboplatin for the first-line treatment of adult patients with metastatic non-squamous NSCLC with no EGFR or ALK genomic tumor aberrations	✖	**✔**
Cemiplimab (Sanofi)	EMPOWER Lung 1 (46)	As monotherapy for the first-line treatment of patients with NSCLC whose tumors have high PD-L1 expression (Tumor Proportion Score ≥50%), with no EGFR, ALK or ROS1 aberrations, and disease that is locally advanced where patients are not candidates for surgical resection or definitive chemoradiation, or metastatic	✖	**✔**
Nivolumab (BMS)	CheckMate-227 (47)	In combination with ipilimumab as first-line treatment for adult patients with metastatic NSCLC expressing PD-L1 (≥1%) as determined by an FDA-approved test, with no EGFR or ALK genomic tumor aberrations	✖	**✔**
	CheckMate-9LA (45)	In combination with ipilimumab and 2 cycles of platinum-doublet chemotherapy for the first-line treatment of adult patients with metastatic or recurrent NSCLC with no EGFR or ALK genomic tumor aberrations	✖	**✔**
Camrelizumab (HengRui)	CameL (48, 49)	In combination with pemetrexed and carboplatin as first-line treatment for patients with unresectable locally advanced or metastatic non-squamous NSCLC, with no EGFR or ALK genomic tumor aberrations	**✔**	✖
Tislelizumab (BeiGene)	RATIONALE-307 (50, 51)	In combination with carboplatin and either paclitaxel or albumin-bound paclitaxel, as a first-line treatment for patients with metastatic squamous NSCLC	**✔**	✖
	RATIONALE-304 (52)	In combination with chemotherapy as a first-line treatment for patients with advanced non-squamous NSCLC	**✔**	✖
Sintilimab (Innovent)	ORIENT-11 (53)	In combination with pemetrexed and platinum chemotherapy as first-line treatment of patients with advanced or metastatic non-squamous NSCLC, with no EGFR or ALK genomic tumor aberrations	**✔**	✖
	ORIENT-12 (54)	In combination with gemcitabine and platinum-based chemotherapy for the first-line treatment of patients with unresectable advanced or recurrent squamous cell NSCLC	**✔**	✖

FDA, US food and drug administration; NMPA, China National Medical Products Administration; NSCLC, non-small cell lung cancer; PD-L1, programmed death ligand-1.

a The FDA approval included pembrolizumab in combination with carboplatin and paclitaxel or albumin-bound paclitaxel.

## ICI as First-Line Therapy For Advanced NSCLC

### Clinical Trials of ICI Monotherapy

Mounting evidence from randomized controlled trials has confirmed that, compared with platinum-based chemotherapy, ICIs provide better clinical outcomes in patients with advanced NSCLC (**Table 2**) (9, 67, 68). Some of the trials were conducted only in China, and some were international multicenter clinical trials that involved Chinese sites (69). The KEYNOTE-001 trial was the first to show that previously untreated patients with advanced or metastatic NSCLC benefited from pembrolizumab monotherapy. In treatment-naive patients, pembrolizumab monotherapy provided a 5-year overall survival (OS) rate of 23.2%. The 5-year OS rate was 29.6% for treatment-naive patients with PD-L1-high (tumor proportion score [TPS] ≥ 50%) NSCLC and 15.7% for treatment-naive patients with PD-L1-low (TPS 1%–49%) NSCLC (70). The subsequent randomized phase III trial KEYNOTE-024 showed that, compared with platinum-based chemotherapy, first-line treatment with pembrolizumab provided better progression-free survival (PFS), OS, and objective response rate (ORR) in previously untreated patients with metastatic, PD-L1-high (TPS ≥ 50%) NSCLC (median PFS, 10.3 vs. 6.0 months; median OS, 26.3 vs. 13.4 months; ORR, 44.8% vs. 27.8%; updated 5-year OS rate, 31.9% vs. 16.3%) (60, 61). The findings of KEYNOTE-024 led to the FDA approval of pembrolizumab as a first-line treatment for selected patients with metastatic *EGFR*/*ALK* wild-type NSCLC and with PD-L1 TPS ≥ 50% (71).

**Table 2:** Summary of key phase III trials using immune checkpoint inhibitors as first-line monotherapy or combination therapy for NSCLC.

Study	Cohort size	PD-L1 expression	Treatment arms	NSCLC histological type	Median PFS (months)	Median OS (months)	ORR (%)	OS rate (%)^a^	Ref
**ICI monotherapy**									
KEYNOTE-024	305	PD-L1 ≥ 50%	Pembrolizumab vs. platinum-based chemotherapy	NSQ+SQ	10.3 vs. 6.0^b^	26.3 vs. 13.4	44.8 vs. 27.8	31.9 vs. 16.3 (5-year rate)	(60, 61)
KEYNOTE-042	1274	PD-L1 ≥ 1%	Pembrolizumab vs. platinum-based chemotherapy	NSQ+SQ	5.4 vs. 6.5	16.4 vs. 12.1^b^	27.0 vs. 27.0	25 vs. 17 (3-year rate)	(34, 35)
		PD-L1 ≥ 20%			6.2 vs. 6.6	18.0 vs. 13.0^b^	33 vs. 29	28 vs. 19 (3-year rate)	
		PD-L1 ≥ 50%			7.1 vs. 6.4	20.0 vs. 12.2^b^	39 vs. 32	31 vs. 18 (3-year rate)	
KEYNOTE-042 China extension	262	PD-L1 ≥ 1%	Pembrolizumab vs. platinum-based chemotherapy	NSQ+SQ	6.3 vs. 6.7	20.2 vs. 13.5^b^	31.3 vs. 24.6	43.8 vs.	(36)
		PD-L1 ≥ 20%			6.2 vs. 7.2	21.9 vs. 13.5^b^	33.7 vs. 24.3	28.2 (2-year rate)	
		PD-L1 ≥ 50%			8.3 vs. 6.5	24.5 vs. 13.8^b^	40.3 vs. 24.3	46.5 vs.	
								28.7 (2-year rate)	
								50.0 vs.	
								29.7 (2-year rate)	
IMpower110	554	TC 1/2/3/and IC 1/2/3 (analysis in TC 3/IC 3)	Atezolizumab vs. chemotherapy	NSQ+SQ	8.1 vs. 5.0	20.2 vs. 14.7^b^	38.3 vs. 28.6		(40, 41)
EMPOWER Lung 1	563	PD-L1 ≥ 50%	Cemiplimab vs. chemotherapy	NSQ+SQ	8.2 vs. 5.7^b^	NR vs. 14.2^b^	39 vs. 20		(46)
CheckMate 026	423	PD-L1 ≥ 1% (primary endpoint assessed in PD-L1 ≥ 5%)	Nivolumab vs. chemotherapy	NSQ+SQ	4.2 vs. 5.9^b^	14.4 vs. 13.2	26.0 vs. 33.0		(62)
**ICI plus chemotherapy**									
KEYNOTE-189	616	PD-L1 unselected	Pembrolizumab/PC vs. PC	NSQ	9.0 vs. 4.9^b^	22.0 vs. 10.7^b^	48.0 vs. 19.4	31.3 vs. 17.4 (3-year rate)	(33)
IMpower130	723	PD-L1 unselected	Atezolizumab/chemotherapy vs. chemotherapy	NSQ	7.0 vs. 5.5^b^	18.6 vs. 13.9^b^	49.2 vs. 31.9	39.6 vs. 30.0 (2-year rate)	(44, 45)
CameL	412	PD-L1 unselected	Camrelizumab/ chemotherapy vs. chemotherapy	NSQ	11.3 vs. 8.3^b^	27.9 vs. 20.5	60.5 vs. 38.6		(48, 49)
ORIENT-11	397	PD-L1 unselected	Sintilimab/ chemotherapy vs. chemotherapy	NSQ	8.9 vs. 5.0^b^	Not reported	51.9 vs. 29.8		(53)
ORIENT-12	357	PD-L1 unselected	Sintilimab/chemotherapy vs. chemotherapy	SQ	5.5 vs. 4.9^b^	Not reached	44.7 vs. 35.4		(54)
RATIONALE 304	332	PD-L1 unselected	Tislelizumab/chemotherapy vs. chemotherapy	NSQ	9.7 vs. 7.6^b^	Not reached	57.4 vs. 36.9		(52)
KEYNOTE-407	559	PD-L1 unselected	Pembrolizumab/chemotherapy vs. carboplatin/ nab-paclitaxel	SQ	8.0 vs. 5.1^b^	17.1 vs. 11.6^b^	62.6 vs. 38.4	29.7 vs. 18.2 (3-year rate)	(37, 38)
KEYNOTE-407 China extension	125	PD-L1 unselected	pembrolizumab/chemotherapy vs. carboplatin/ (nab-) paclitaxel	SQ	8.3^b^ vs. 4.2	17.3 vs. 12.6^b^	78.5 vs. 41.7		(63)
CameL-sq	390	PD-L1 unselected	Camrelizumab/ chemotherapy vs. chemotherapy	SQ	8.5 vs. 4.9^b^	NR vs. 14.5	64.8 vs. 36.7		(64)
RATIONALE 307	360	PD-L1 unselected	Tislelizumab/PC or nab-PC vs. PC	SQ	7.6, 7.6 vs. 5.5^b^	Not reported	73,75 vs. 50		(50, 51)
GEMSTONE-302	479	PD-L1 unselected	Sugemalimab/chemotherapy vs. chemotherapy	NSQ+SQ	7.8 vs. 4.9^b^	Not reported	61.4 vs. 39.2		(65)
**ICI plus chemotherapy and antiangiogenic therapy**									
IMpower150	1202	Analysis in PD-L1 unselected, EGFR/ALK	Chemotherapy plus bevacizumab ± atezolizumab	NSQ	8.3 vs. 6.8^b^	19.5 vs. 14.7^b^	63.5 vs. 48.0	43.4 vs. 33.7 (2-year rate)	(42, 43)
		WT (study included any EGFR/ALK status)							
**Dual ICI**									
KEYNOTE-598	568	PD-L1 ≥ 50%	Pembrolizumab ± ipilimumab	NSQ+SQ	8.2 vs. 8.4^b^	21.4 vs. 21.9^b^	45.4 vs. 45.4		(66)
CheckMate 227 part 1a	1189	PD-L1 ≥ 1%	Ipilimumab/ nivolumab vs. chemotherapy	NSQ+SQ	5.1 vs. 5.6^b^	17.1 vs. 14.9^b^	35.9 vs. 30.0	40.0 vs. 32.8 (2-year rate)	(47)
**Dual ICI plus chemotherapy**									
CheckMate 9LA	719	PD-L1 unselected	Ipilimumab/ nivolumab/chemotherapy vs. chemotherapy	NSQ+SQ	6.8 vs. 5.0	15.6 vs. 10.9^b^	37.7 vs. 25.1		(45)

IC, tumor-infiltrating immune cells covering % of the tumor area; ICI, immune checkpoint inhibitor; NSQ, nonsquamous cell carcinoma; OS, overall survival; PFS, progression-free survival; PC, carboplatin/pemetrexed; ORR, objective response rate; SQ, squamous cell carcinoma; TC, expression of PD-L1 on tumor cells.

a Only survival rates ≥ 2 years are shown.

b Primary endpoint.

The clinical benefit of first-line pembrolizumab monotherapy in patients with PD-L1-positive and *EGFR/ALK* wild-type advanced NSCLC was confirmed in the multicenter, randomized, open-label, controlled phase III study KEYNOTE-042 (34). In this study, previously untreated patients with advanced NSCLC harboring no *EGFR* mutation or *ALK* rearrangement were stratified at randomization based on region of enrollment, performance status, histology, and PD-L1 expression (34). The results demonstrated that, compared with chemotherapy, pembrolizumab monotherapy significantly prolonged OS in patients with locally advanced (stage III) or metastatic NSCLC, with the greatest treatment effect seen in PD-L1-high patients (PD-L1 ≥ 50%: 20.0 vs. 12.2 months; HR, 0.68; 95% CI, 0.57–0.82; PD-L1 ≥ 20%: 18.0 vs. 13.0 months; HR, 0.75; 95% CI, 0.64–0.88; PD-L1 ≥ 1%: 16.4 vs. 12.1 months; HR, 0.80; 95% CI, 0.71–0.90) (34, 35). Based on these findings, FDA approval of pembrolizumab monotherapy as a first-line treatment was extended to patients with locally advanced (stage III) or metastatic, *EGFR*/*ALK* wild-type NSCLC and with PD-L1 TPS ≥ 1% as determined by an FDA-approved test (72).

In an extension of the KEYNOTE-042 study in a Chinese cohort, pembrolizumab monotherapy significantly improved OS compared with chemotherapy alone in patients with PD-L1-positive NSCLC with a PD-L1 TPS ≥ 50% (median OS, 24.5 vs 13.8 months; HR, 0.63; 95% CI, 0.43–0.94), TPS ≥ 20% (median OS, 21.9 vs 13.5 months; HR, 0.66; 95% CI, 0.47–0.92), and TPS ≥ 1% (median OS, 20.2 vs 13.5 months; HR, 0.67; 95% CI, 0.50–0.89) (36). The results of the KEYNOTE-042 China extension study were crucial for the approval of pembrolizumab monotherapy as a first-line treatment of advanced PD-L1-positive NSCLC in China (36, 56).

Atezolizumab is an anti-PD-L1 monoclonal antibody recently approved by the FDA and the NMPA for the first-line treatment of patients with metastatic, PD-L1 high expression nonsquamous or squamous NSCLC harboring no *EGFR* mutation or ALK rearrangement. This approval was based on the findings of IMpower110 (NCT02409342), an international, randomized, open-label trial comparing the efficacy of atezolizumab to that of platinum-based chemotherapy in chemotherapy-naïve patients with stage IV NSCLC expressing PD-L1 on ≥ 1% of tumor cells (TC ≥ 1%) or tumor-infiltrating immune cells (IC ≥ 1%). In patients with high PD-L1 expression (TC ≥ 50% or IC ≥ 10%), atezolizumab provided longer OS (median, 20.2 vs. 13.1 months; stratified HR, 0.59; 95% CI, 0.40−0.89) and PFS (median, 8.1 vs. 5.0 months; stratified HR, 0.63; 95% CI, 0.45-0.88) than chemotherapy (40, 73, 74). An updated analysis of IMpower110 found no difference in OS for atezolizumab versus chemotherapy for patients with high or intermediate (≥ 5% TC or IC) PD-L1 expression (n = 328; median, 19.9 vs 16.1 months; HR, 0.87; 95% CI, 0.66−1.14); *P* = 0.3091) and endpoints were therefore not formally tested in patients with any PD-L1 expression (41). However, an exploratory analysis showed that the OS benefit observed for atezolizumab versus chemotherapy in patients with high PD-L1 expression (TC ≥ 50% or IC ≥ 10%) was maintained after 17 months’ additional follow-up (median, 20.2 vs 14.7 months; HR, 0.76; 95% CI, 0.54−1.09) (41).

Cemiplimab is an FDA-approved monoclonal antibody targeting PD-1 for the first-line treatment of patients with locally advanced or metastatic, PD-L1-high nonsquamous and squamous NSCLC harboring no *EGFR*/*ALK*/*ROS1* alterations. The global, multicenter, open-label phase III study EMPOWER-Lung 1 investigated the efficacy and safety of cemiplimab in treatment-naïve patients with advanced squamous or non-squamous NSCLC expressing high PD-L1 levels (TPS ≥ 50%). Preliminary data showed that compared with platinum-doublet chemotherapy, cemiplimab provided superior OS (median, NR vs. 14.2 months; HR, 0.57; 95% CI, 0.42–0.77) and PFS (median, 8.2 vs. 5.7 months; HR, 0.54; 95% CI, 0.43–0.68) in patients with NSCLC expressing high PD-L1 levels. The toxicity profile of cemiplimab was also superior to that of platinum-doublet chemotherapy (46).

No benefit has been observed with nivolumab monotherapy in this setting. The open-label, randomized, phase III clinical study CheckMate 026 compared first-line nivolumab with chemotherapy in 423 previously untreated patients with PD-L1-positive (PD-L1 ≥ 5%) NSCLC. The median PFS (primary endpoint) in nivolumab-treated patients was 4.2 months, not significantly different from the median PFS of 5.9 months in patients treated with chemotherapy (HR, 1.15; 95% CI, 0.91–1.45; *P* = 0.25). The OS (secondary endpoint) was similar in the nivolumab and chemotherapy groups (median, 14.4 vs. 13.2 months; HR, 1.02; 95% CI, 0.80–1.30) (62).

### Clinical Trials of ICI Combination Therapy

Data suggests that PD-L1 is upregulated in tumors during chemotherapy (75, 76). Additionally, chemotherapy enhances the presentation of tumor antigens and reduces the number of different Treg subsets (77–79). Hence, the efficacy of first-line PD-1/PD-L1 inhibitors combined with chemotherapy has been evaluated in patients with NSCLC. In the phase II KEYNOTE-021 trial, data from Cohort G showed that first-line pembrolizumab combined with chemotherapy provided a higher ORR (58% vs. 33%) and longer PFS (median, 24.5 vs. 9.9 months), and a long-term follow up (median 49.4 months) revealed a longer OS (median, 34.5 vs. 21.1 months; HR, 0.71; 95% CI, 0.45‒1.12), versus chemotherapy alone in patients with advanced *EGFR*/*ALK* wild-type, nonsquamous NSCLC (80–82). A subsequent phase III study (KEYNOTE-189) of patients with metastatic *EGFR*/*ALK* wild-type nonsquamous NSCLC has shown that, compared with pemetrexed and platinum-based chemotherapy alone, first-line pembrolizumab plus chemotherapy conferred better OS (median, 22.0 vs. 10.7 months; HR, 0.56; 95% CI, 0.45–0.70), 3-year OS rate (31.3 vs. 17.4%) and PFS (median, 9.0 vs. 4.9 months; HR, 0.48; 95% CI, 0.40–0.58); ORR (48.0% vs. 19.4%) was also extended by 28.6% in patients who received combination therapy (33). Similarly, in the phase III KEYNOTE-407 trial, the combination of pembrolizumab with chemotherapy (carboplatin and paclitaxel or nab-paclitaxel) provided longer OS (median, 17.1 vs. 11.6 months; HR, 0.71; 95% CI, 0.58–0.88), 3-year OS rate (29.7 vs. 18.2%) and PFS (median, 8.0 vs. 5.1 months; HR, 0.57; 95% CI, 0.47–0.69) compared with chemotherapy alone in patients with metastatic squamous NSCLC (37). In the KEYNOTE-407 China extension study, the efficacy of pembrolizumab plus chemotherapy (carboplatin and paclitaxel) in PD-L1-unselected patients with metastatic squamous NSCLC was compared to that of chemotherapy alone. Compared with chemotherapy alone, the combination therapy provided superior PFS (median, 8.3 vs. 4.2 months; HR; 0.32 95% CI, 0.21–0.49), OS (median, 17.3 vs. 12.6 months; HR; 0.44 95% CI, 0.24–0.81), and ORR (78.5% vs. 41.7%) (63). Patients in the KEYNOTE-189 and KEYNOTE-407 studies were not selected based on PD-L1 expression levels (37). Based on the findings of these studies, the NMPA has approved pembrolizumab as a first-line treatment in combination with pemetrexed and platinum chemotherapy for metastatic nonsquamous NSCLC (*EGFR*/*ALK* wild-type) and in combination with carboplatin and paclitaxel for metastatic squamous NSCLC (83).

Other ICIs have also been investigated as combination therapy for the first-line treatment of advanced NSCLC. Camrelizumab was a newly developed domestic anti-PD-1 monoclonal antibody approved by the NMPA for first-line use in patients with nonsquamous NSCLC in China. CameL (NCT03134872) was a randomized, open-label, phase III trial evaluating the efficacy of camrelizumab combined with carboplatin and pemetrexed in Chinese treatment-naïve patients with advanced nonsquamous NSCLC without EGFR and ALK alterations. Compared with chemotherapy alone, combination therapy significantly prolonged PFS (median, 11.3 vs. 8.3 months; HR; 0.60 95% CI, 0.45–0.79). Camrelizumab combined with chemotherapy also prolonged OS (median, 27.9 vs. 20.5 months; HR; 0.73 95% CI, 0.55–0.96), which was the secondary endpoint of the study (48). The efficacy of first-line camrelizumab combined with carboplatin and paclitaxel was also investigated in patients with advanced squamous NSCLC in the randomized, double-blind, phase III clinical study CameL-sq (NCT03668496). In this cohort, the combination of camrelizumab with chemotherapy significantly prolonged PFS, which was the primary endpoint of the study. Specifically, the median PFS was 8.5 months in patients treated with camrelizumab plus chemotherapy and 4.9 months in patients treated with placebo plus chemotherapy (HR, 0.37; 95% CI, 0.29–0.47). OS was not reached in the camrelizumab plus chemotherapy arm and was 14.5 months in the placebo plus chemotherapy arm (64).

The multicenter randomized trial IMpower130 (NCT02367781) investigated the efficacy of atezolizumab combined with standard chemotherapy in treatment-naïve patients with metastatic nonsquamous NSCLC. The combination of atezolizumab with nab-paclitaxel and carboplatin provided longer OS (median, 18.6 vs. 13.9 months; HR; 0.79 95% CI, 0.64–0.98) and PFS (median, 7.0 vs. 5.5 months; HR; 0.64 95% CI, 0.54–0.77) than chemotherapy alone in patients without EGFR and ALK alterations (44). The multicenter, randomized, phase III trial IMpower150 (NCT02366143) evaluated the antitumor effects of first-line atezolizumab combined with chemotherapy (carboplatin plus paclitaxel) and antiangiogenic therapy (bevacizumab) in patients with metastatic nonsquamous NSCLC. Compared with chemotherapy plus bevacizumab, the combination of atezolizumab with chemotherapy and bevacizumab provided longer OS (median, 19.5 vs. 14.7 months; HR; 0.80 95% CI 0.67–0.95) and PFS (median, 8.3 vs. 6.8 months; HR; 0.62 95% CI 0.52–0.74) in patients with *EGFR/ALK* wild-type NSCLC (42, 43).

The efficacy of first-line dual immunotherapy alone or in combination with chemotherapy has also been evaluated. In part 1a of the phase III CheckMate 227 trial, previously untreated patients with advanced NSCLC (*EGFR*/*ALK* wild-type) and PD-L1 ≥1% were randomized (1:1:1) to nivolumab plus ipilimumab, nivolumab alone or chemotherapy alone (47). In part 1b, patients with PD-L1 <1% were randomly assigned to nivolumab plus ipilimumab, nivolumab plus chemotherapy, or chemotherapy alone. The results from CheckMate 227 part 1a showed that combination immunotherapy with nivolumab plus ipilimumab led to a better OS and moderately higher PFS and ORR than chemotherapy alone (median OS: 17.1 vs. 14.9 months; HR, 0.79; 97.72% CI, 0.65–0.96; median PFS: 5.1 vs. 5.6 months; HR, 0.82; 95% CI, 0.69–0.97; ORR: 35.9% vs. 30.0%); in patients with a PD-L1 expression level ≥1%. In the global phase III study CheckMate 9LA, treatment-naïve patients with advanced NSCLC were randomly assigned to receive nivolumab plus ipilimumab combined with two cycles of chemotherapy or chemotherapy alone. Compared with chemotherapy alone, the combination of nivolumab plus ipilimumab with two cycles of chemotherapy significantly improved the OS of previously untreated patients with advanced NSCLC [median OS: 15.6 vs. 10.9 months; HR, 0.66; 95% CI, 0.55–0.80 (84)]. However, these studies did not include Chinese patients.

### Ongoing Clinical Trials

Currently, various domestic and imported ICIs are under clinical investigation in China for use in the first-line treatment of advanced NSCLC combined with chemotherapy or anti-VEGF antibody or as dual ICI therapy. The results of key phase III trials are summarized in **Table 1**. Trials of sintilimab, tislelizumab and sugemalimab (e.g., ORIENT-11, ORIENT-12, RATIONALE 304, RATIONALE 307, GEMSTONE-302) have already reported their interim analysis of the primary endpoints and confirmation of OS benefit needs a longer follow-up. Other studies (e.g., IMpower151, LEAP 006, LEAP 007) are still under investigation.

Sintilimab is a humanized anti-PD-1 monoclonal antibody approved by the NMPA for the first-line treatment of patients with nonsquamous and squamous NSCLC (85, 86). ORIENT-11 (NCT03607539) was a randomized, double-blind, phase III study investigating the use of sintilimab combined with pemetrexed and platinum chemotherapy in patients with locally advanced or metastatic nonsquamous NSCLC; patients were stratified by PD-L1 expression (TPS ≥ 1% or < 1%). Analysis of the primary endpoint (PFS) showed that, compared with chemotherapy alone, sintilimab plus chemotherapy prolonged PFS (median, 8.9 vs. 5.0 months; HR; 0.482 95% CI, 0.362–0.643) (53). ORIENT-12 (NCT03629925) was a randomized, double-blind, phase III study assessing the efficacy and safety of sintilimab combined with gemcitabine and platinum-based chemotherapy in previously untreated patients with advanced or recurrent squamous NSCLC. An updated analysis after a 12.9-month median follow-up revealed a median PFS of 5.5 months in the combination treatment group vs. 4.9 months in the chemotherapy alone group (HR, 0.536; 95% CI, 0.422–0.681). Toxicities were similar in the two treatment groups (54).

Tislelizumab is a humanized anti-PD-1 monoclonal antibody approved by the NMPA for use in patients with nonsquamous or squamous NSCLC. The randomized, open-label, phase III RATIONALE 304 (NCT03663205) trial assessed the efficacy and safety of tislelizumab plus chemotherapy (pemetrexed and carboplatin or cisplatin) versus chemotherapy alone in treatment-naïve patients with advanced nonsquamous NSCLC (52). The preliminary results of this study revealed a significantly longer PFS with tislelizumab versus chemotherapy (median, 9.7 vs. 7.6 months; HR, 0.645; 95% CI, 0.462–0.902). Furthermore, the ongoing RATIONALE 307 (NCT03594747) study is a randomized, open-label, phase III trial investigating the first-line use of tislelizumab combined with chemotherapy (carboplatin and paclitaxel or nab-paclitaxel) in patients with advanced squamous NSCLC. Preliminary data from RATIONALE 307 showed that, compared with chemotherapy alone, the combination of tislelizumab plus chemotherapy significantly improved median PFS and ORR in patients with squamous NSCLC (50, 67).

Sugemalimab (CS1001) is an investigational monoclonal antibody targeting human PD-L1, and GEMSTONE-302 is a randomized, double-blind, phase III study evaluating the efficacy and safety of sugemalimab plus platinum-based chemotherapy in previously untreated patients with advanced squamous or nonsquamous NSCLC. Preliminary data suggest that first-line sugemalimab combined with chemotherapy may prolong patient survival (ITT: median PFS 7.82 vs. 4.90 months [HR, 0.50 (95% CI, 0.39-0.64)]; nonsquamous: median PFS 8.57 vs. 5.16 months [HR, 0.66]; squamous: median PFS 7.16 vs. 4.70 months, [HR, 0.33]). ORRs were 61.4% (56.2% for nonsquamous, 69.0% for squamous) in the sugemalimab plus chemotherapy group and 39.2% (34.7% for nonsquamous, 46.0% for squamous) in the chemotherapy alone group (65).

### PD-L1 Expression Level as a Predictor of Response to First-Line Immunotherapy of NSCLC

Mounting clinical evidence suggests that in NSCLC and other immunogenic malignancies, PD-L1 expression levels in tumor cells are strongly associated with response to PD-1/PD-L1 inhibitors. Currently, PD-L1 expression in tumor cells is the most important predictive biomarker of response to ICIs. In NSCLC, high PD-L1 expression levels in tumor cells have been associated with favorable response to immunotherapy and prolonged survival after first-line therapy with ICIs (87). Given the use of pembrolizumab as first-line treatment for certain patients with NSCLC, PD-L1 expression testing and *EGFR*/*ALK* status testing are recommended at the first diagnosis of patients with advanced NSCLC in China, according to the 2020 Chinese Society of Clinical Oncology (CSCO) guidelines (55, 56).

PD-L1 IHC 22C3 pharmDx, a PD-L1 antibody reagent, was approved by the NMPA as a qualitative immunohistochemical assay to assess PD-L1 expression in formalin-fixed, paraffin-embedded NSCLC biopsy/surgical pathology specimens using a monoclonal mouse anti-PD-L1 antibody (clone 22C3, code M3653, Dako) on May 8, 2020 (88). In China, PD-L1 detection using PD-L1 IHC 22C3 pharmDx is currently used to identify patients with NSCLC suitable for treatment with pembrolizumab (55). PD-L1 IHC 22C3 pharmDx was also licensed in the US and Europe as a companion diagnostic test for pembrolizumab treatment of NSCLC (88). The kit is used to assess the TPS for PD-L1 to determine the proportion of viable cancer cells showing partial or complete PD-L1 membrane staining (55, 88). Typically, specimens with TPS < 1% are considered as PD-L1-negative, whereas samples with TPS ≥ 1% are considered as PD-L1-positive; TPS 1%–49% indicates a low PD-L1 expression and TPS ≥ 50% indicates a high PD-L1 expression (55, 88). Similarly, VENTANA PD-L1 (SP142) Assay has been approved by the FDA as a companion diagnostic test to identify patients with NSCLC who are most likely to benefit from atezolizumab. The test can be used to determine PD-L1 expression based on both tumor cell staining (TC score) and tumor-infiltrating immune cell staining (IC score) (58, 89).

## Adverse Reactions Related to ICIs

Immune checkpoints are key molecules in maintaining immune homeostasis and preventing tissue damage due to excessive or prolonged immune system activation. Thus, ICIs can induce various immune-related adverse events (IRAEs) (68). The most commonly affected systems in patients experiencing IRAEs are the gastrointestinal, dermatological, and endocrine systems (68). The most common side effects of ICIs are rash and mucosal irritation, occurring in nearly 50% of patients (27). Diarrhea is also common among patients treated with ICIs, especially among those receiving CTLA-4 inhibitors. However, most adverse events are mild or moderate, with grade 3 or 4 diarrhea occurring in less than 10% of patients receiving ICIs (27). Autoimmune hepatotoxicity, indicated by an increase in aspartate aminotransferase levels, is a severe yet less common side effect occurring in less than 10% of patients receiving PD-1/PD-L1 inhibitors (27). Pulmonary, neurologic, hematologic, and cardiac adverse effects have also been reported. In a real-world retrospective analysis of Chinese patients with NSCLC who received ICIs, Chen et al. (90) reported a 46.4% incidence of IRAEs, most of which were grade 2; grade 3 or 4 IRAEs occurred in 9.4% of patients. The study also revealed that treatment interruption due to severe IRAEs (myocarditis, pneumonia, and grade 4 bullous lung disease) was relatively rare (90).

## Challenges and Future Directions

ICIs have led to remarkable improvements in survival outcomes in patients with advanced NSCLC compared with conventional chemotherapy. The long-term survival benefit of ICIs results in characteristic “long tail” survival curves. Notwithstanding the promising safety and efficacy of pembrolizumab and other ICIs for the first-line treatment of NSCLC, in general, a proportion of patients will not achieve a response to ICI therapy. Future clinical studies are therefore required to identify strategies to expand the proportion of patients who benefit from treatment with ICIs through identification of patients likely to respond to ICI therapy, developing strategies to overcome immunotherapy resistance, exploring the potential benefit of ICIs for patients with EGFR-mutant NSCLC and better understanding the safety profile of ICI therapy (91, 92).

Further research is required to identify and validate factors predicting response to pembrolizumab and other ICIs, and this would facilitate the identification of patients most likely to benefit from ICB alone or in combination with chemotherapy (93). Given that resistance to ICIs is common, approaches to accurately predict ICI response will spare non-responders from the unnecessary adverse effects of immunotherapy (55). Factors including gut microbiota (28) and tumor mutation burden (TMB) (94) have been associated with response to ICI in preliminary studies but require further investigation and validation in large cohort studies. In addition, in China, there are many challenges for PD-L1 testing, and the clinical application of PD-L1 testing is still at an early stage (55, 56, 83). The establishment of standardized guidelines on PD-L1 testing will enhance the clinical significance of PD-L1 testing as a means to predict response to PD-1/PD-L1 inhibitors (55). Given the high intertumoral and intratumoral heterogeneity in PD-L1 expression, future studies are also warranted to identify other robust biomarkers (e.g., serum biomarkers, neutrophil-to-lymphocyte ratio) for predicting response to ICIs (83, 95–97). These biomarkers should comprehensively capture the immunological status of the tumor based on multiple components of the tumor microenvironment rather than the expression of a single checkpoint molecule (95, 96, 98).

Tumor immune escape and development of resistance to immunotherapies remain significant challenges limiting the clinical benefit of ICIs (91). Therefore, the development of strategies to target mechanisms driving immune tolerance and immunotherapy resistance will maximize the clinical benefit of pembrolizumab and other ICIs, as well as facilitate long-lasting responses. Promising actionable targets driving immune tolerance are regulatory T cells (Tregs), M2 phenotype tumor-associated macrophages (TAMs), and myeloid-derived suppressor cells (MDSCs) (99). Immunomodulatory pathways include IFNγ, mitogen-activated protein kinase (MAPK), and WNT/β-catenin pathways. The potential clinical benefit of treatment regimens combining ICIs, chemotherapy, radiotherapy, targeted therapies, and antiangiogenic therapies should be further explored as promising ways to combat resistance of NSCLC to ICIs (100).

The potential use of ICIs to treat patients with *EGFR*-mutant NSCLC merits further investigation (101, 102). The relationship between PD-L1/PD-1 expression levels and *EGFR* mutation status, as well as the interaction between the PD-1/PD-L1 and EGFR pathways, are poorly understood (56, 103–106). Notably, a large−scale, multicenter, real−world study in China identified a significant association between PD-L1 expression levels and *EGFR* mutation and *ALK* rearrangement (107). Future Chinese studies are critical to addressing this issue given the higher *EGFR* mutation rates in Chinese patients with NSCLC compared with their Western counterparts (approximately 28% in unselected Chinese patients with NSCLC vs. 17% in Western populations) (28, 108, 109).

As most clinical trials investigating the use of ICIs are still ongoing, more clinical data from large-cohort, long-term studies are required to elucidate the adverse effects associated with ICB, especially in elderly (≥ 65 years old) and immunocompromised patients. In China, the management of IRAEs is based on Management of Immunotherapy-Related Toxicities guidelines, version 1.2019, although NCCN Management of Immunotherapy-Related Toxicities guidelines version 1.2020 is also used (56, 110). The further identification of risk factors associated with AEs during ICB is critical for the prevention and early management of IRAEs. In addition, there are currently limited data on the efficacy and safety of ICIs in special patient populations including older patients, patients with hepatitis virus or HIV infections, and those with poor performance status, brain or liver metastases, or autoimmune diseases (111, 112). These patient groups are often excluded from clinical trials and in China further data are required to support the establishment of treatment guidelines for such cohorts of patients with NSCLC.

## Discussion

Despite recent advances in the treatment of NSCLC using targeted therapies and immunotherapies, NSCLC remains the leading cause of cancer-related death in China. Therefore, novel agents and combination treatment strategies are urgently required to improve the survival outcomes of patients with advanced NSCLC. ICIs have revolutionized the treatment of NSCLC and other solid malignancies in China and worldwide. Clinical data suggest that pembrolizumab is a promising immunotherapeutic agent for the first-line treatment of patients with advanced NSCLC and has the most NMPA-approved indications. Notably, pembrolizumab is the only ICI providing an impressive 5-year survival rate in the first-line treatment of advanced NSCLC. In China, pembrolizumab has gained regulatory approval for use as the first-line treatment for NSCLC, either as monotherapy or in combination with chemotherapy. As numerous other domestic and imported ICIs are under extensive clinical investigation as monotherapy or combination therapy, more options will become available for the treatment of Chinese patients with advanced NSCLC.

Additionally, PD-L1 IHC 22C3 pharmDx is currently the only licensed NSCLC companion diagnostic in China, which could aid in selecting appropriate patients for pembrolizumab treatment and optimize the pembrolizumab regimen to maximize its clinical benefits. This review provides an in-depth insight into recent advances in NSCLC immunotherapy and discusses future perspectives on the use of ICIs for the first-line treatment of NSCLC in China. As such, this and future comprehensive reviews of current and emerging immunotherapies for the first-line treatment of NSCLC are pivotal for the optimal management of NSCLC in China and for maximizing the clinical efficacy of ICIs in treatment-naïve patients with advanced NSCLC.

## Author Contributions

AX, JW, and CZ provided the concept for the review, and also contributed substantive suggestions for revision or critically reviewed subsequent iterations of the manuscript. All authors reviewed and approved the final version of the paper and agree to be accountable for all aspects of the work in ensuring that questions related to the accuracy or integrity of any part of the work are appropriately investigated and resolved.

## Funding

This review was funded by MSD China.

## Conflict of Interest

JW was employed by MSD China. CZ reports personal fees from Lily China, Sanofi, BI, Roche China, MSD, Qilu, Hengrui, Innovent Biologics, C-stone, LUYE Pharma, TopAlliance Biosciences Inc, Amoy Diagnostics, outside the submitted work.

The remaining author declares that the research was conducted in the absence of any commercial or financial relationships that could be construed as a potential conflict of interest.

The authors declare that this study received funding from MSD China. The funder had the following involvement in the study: developing the concept of the review, the writing of this article and the decision to submit it for publication.

## Publisher’s Note

All claims expressed in this article are solely those of the authors and do not necessarily represent those of their affiliated organizations, or those of the publisher, the editors and the reviewers. Any product that may be evaluated in this article, or claim that may be made by its manufacturer, is not guaranteed or endorsed by the publisher.
